# Treating Critical Illness: The Importance of First Doing No Harm

**DOI:** 10.1371/journal.pmed.0020167

**Published:** 2005-06-28

**Authors:** Mervyn Singer, Paul Glynne

## Abstract

Singer and Glynne present evidence to suggest that the short- term benefits of many interventions for treating critical illness may camouflage an underlying tendency to cause harm.

The Battle of Trafalgar was a short-lasting but bloody affair. By the end of the afternoon of 21 October 1805, the French/Spanish fleet had lost 4,408 men, and a further 2,545 were wounded while the victorious British force suffered 455 dead and 1,242 wounded [1]. The British flagship HMS Victory, where Admiral Horatio Nelson lost his life to a sniper's bullet, sustained 56 other deaths. The surgeon on board, Dr. William Beatty, also listed 102 wounded sailors who survived the battle or its immediate aftermath [2]. He needed to perform ten amputations, mainly involving the leg, and reported instances of death from gangrene and tetanus. Yet only six of the 102 wounded sailors subsequently died.

This remarkably low mortality rate was mirrored by that sustained by the 13th Light Dragoons during the Battle of Waterloo in 1815. Of 52 privates reported as wounded, only three later died of their wounds [3].

The United States Civil War five decades later was a more protracted but equally bitter conflict, with over 550,000 deaths in the four years of battle [4]. Of note, twice as many troops died from disease, privation, and accidents than died of injuries sustained on the battlefield. Typhus, typhoid fever, mumps, and measles were rife in army camps where poor sanitation, hygiene, and an inadequate diet were the norm. The surgeons in these camps often held their instruments between their teeth, and these tools were only cleaned at the end of the day's operating. At the Battle of Antietam, maize husks were used as bandages. Yet despite the prevailing conditions and lack of aseptic technique, mortality rates were remarkably good. The Union forces kept detailed records and reported a directly attributable mortality rate from battlefield injuries of 14%. The overall mortality rate following amputation was only about 25%, (Table 1) with some patients even surviving hindquarter amputation [5].

**Table 1:**
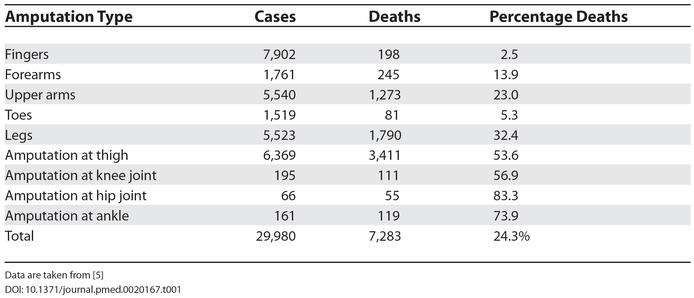
Mortality Rates from Amputations During the US Civil War

Data are taken from [5]

## Then and Now

These survival rates do appear impressive, especially when one considers that they were obtained without antisepsis, antibiotics, blood transfusion, oxygen, and the other paraphernalia of modern medicine, and that the surgeons relied upon rudimentary surgical techniques performed without the assistance or comforts of sophisticated anaesthesia or mechanical ventilation.

Clearly, a direct analogy should not be made to the mortality rates for today's battlefield injuries, which involve far more destructive weaponry, or to those of modern hospital populations, where many patients are elderly and/or immunosuppressed. Nevertheless, it is valid to wonder why even greater improvements in outcome have not been achieved in the last two to three decades despite the huge advances made in medical technology, treatments, and understanding of underlying pathological mechanisms. Barring the 1919 influenza pandemic, Armstrong et al. reported an impressive 22-fold fall in crude mortality rates for infectious diseases in the US between 1900 and 1980 [6]. Yet they also showed how mortality rates (to 1996) have since increased—by 50%—with the septicaemia rate nearly doubling.

While it is true that people are living longer, with an increase in life expectancy from 1980 to 1996 of 2.4 years (from 73.7 to 76.1) [7], these gains are unlikely to be due to advances in hospital medicine. They are far more likely to be due to the contribution of better public health and education, including reductions in environmental pollution, altered eating and smoking habits, and increased exercise.

## The Poor Evidence Base for Many Interventions

An important but generally overlooked consideration is the possibility that superficially attractive, short-term benefits may camouflage an underlying tendency to cause harm. There are high-profile instances where injury is belatedly recognised. A recent example is the increased risk in serious cardiovascular thrombotic events seen in patients taking the anti-inflammatory COX II inhibitor rofecoxib [8]. Clearly, some individuals have suffered, yet the target patient group as a whole has been protected by the much greater surveillance given to a new pharmaceutical compound and the improved likelihood of detecting a major complication. How many long-standing medications, devices, or treatment regimens have been scrutinised to a remotely similar extent?

A fundamental tenet of medical teaching is to first do no harm to our patients. Every decision affecting patient management should thus be judged on the basis of the ratio of likely risks to benefits. Alas, large chunks of perceived wisdom rely on expert opinion, historical practice, and blind acceptance, rather than on an adequate evidence base, to vindicate continued use of a drug, device, or management strategy.

For example, more than half the 50-plus recommendations made in the recent Surviving Sepsis guidelines [9], which have been endorsed and are now being heavily promoted by the US and European critical care societies as a standard of care, were based solely on expert opinion. Many of the other, more highly graded, recommendations relied upon studies with small patient numbers and/or methodological flaws. Of only four Grade A recommendations (i.e., those supported by at least two large, randomised trials with clear-cut results), deep vein thrombosis prophylaxis and ventilator weaning are generic issues for all critically ill patients, while the other two (avoidance of high-dose corticosteroids, and not striving to achieve specified target values of oxygen delivery/consumption when resuscitating patients with fluids and inotropes) were based upon studies performed over a decade ago. The two latter recommendations arose from “negative” studies in which the standards of care at the time were shown to be ineffective [10] or even harmful [11]. This recognition of harm also applies to many of the Grade B recommendations (i.e., supported by one large, randomised trial), for example, the lower threshold of haemoglobin used to trigger blood transfusion [12], or the reduced tidal volumes delivered during mechanical ventilation [13].

## Fashionable Treatments for Critical Illness: Are They Harming Patients?

The major advances of intensive care medicine in the last 20 years have been related more to the recognition and removal of harmful practices rather than to any novel pharmacological or mechanical interventions. It is thus reasonable to question how many currently fashionable strategies may actually prove injurious when submitted to critical examination (Table 2)? This assumes, of course, that the inclination to challenge dogma exists.

**Table 2:**
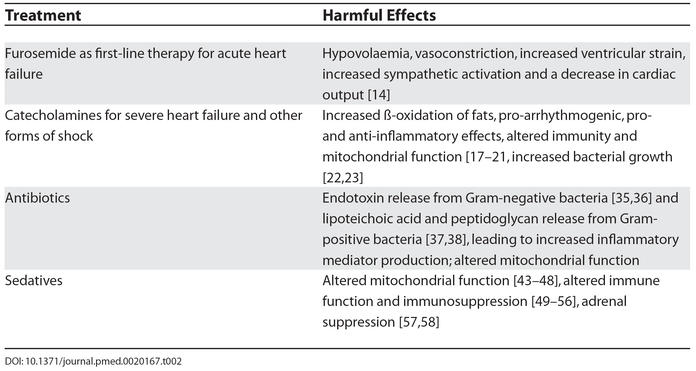
Examples of Fashionable Treatments for Critical Illness That May Cause Harm

A perfect example of this unwavering adherence to an article of faith is the flawed reliance upon furosemide as first-line therapy in the management of patients with acute heart failure. Such patients rarely have intravascular volume overload, yet they are often given a potent loop diuretic, which will frequently result in hypovolaemia, vasoconstriction, increased ventricular strain, and a decrease in cardiac output [14]. This effect is not apparent at the end of the needle, where the initial but short-lived vasodilatation produces symptomatic relief and a transient improvement in haemodynamics. Thus, it is highly convenient to blame the patient's failing heart for any subsequent deterioration without recognition and acceptance of any iatrogenic component.

Recent European Society of Cardiology guidelines for the management of acute heart failure [15] make repeated references to the harmful consequences of diuretic use, emphasising that “secondary effects are frequent and may be life-threatening.” Yet the Task Force of experts still proceeded to make a Class I recommendation for their continued use (i.e., evidence and/or general agreement that a given diagnostic procedure/treatment is beneficial, useful, and effective), with a “B” level of evidence (i.e., data derived from a single, randomised clinical trial or large, nonrandomised studies). Their justification for this grading—which was not actually underpinned by any trial data, either large or randomised—was that “The symptomatic benefits (of diuretics) and their universal clinical acceptance have precluded a formal evaluation in large-scale randomised clinical trials.” Indeed, the single prospective randomised outcome study performed to date (and cited by the guidelines) actually showed the superiority of nitrates over furosemide [16].

The key phrase used above is “universal clinical acceptance”—that is to say, “we believe diuretics work, as we've used them unquestioningly throughout our medical careers, so we can't possibly question this particular shibboleth in a critically objective fashion.” Has there been any consideration of the possibility that the long-term outcome benefits derived from ACE inhibitors may be, at least in part, related to the necessary decrease in diuretic dosing?

In more severe heart failure and other forms of shock associated with low blood pressures and/or low cardiac outputs, there is a conventional reliance upon catecholamines such as dobutamine, norepinephrine, and epinephrine. Yet these inotrope and pressor agents have many effects distant from their cardiovascular actions. They have metabolic effects including increased ß-oxidation of fats; they are pro-arrhythmogenic; they have pro- and anti-inflammatory effects; and they can alter both immunity and mitochondrial function [17–21]. Lyte et al. showed that the use of catecholamine inotropes was associated with significant increases in bacterial growth of Gram-positive [22] and Gram-negative [23] bacteria and in the formation of biofilms [22].

Indeed, all large randomised studies performed to date in patients with chronic heart failure that have compared catecholamines or phosphodiesterase inhibitors (both of which increase cyclic adenosine monophosphate [AMP] levels) against either placebo or another treatment have also shown detriment. Even short-term (1–2 day) infusions of the phosphodiesterase inhibitors milrinone and vesnanrinone significantly worsened six-month outcomes [24,25]. A similar effect has been reported with dobutamine in comparison to the calcium sensitiser levosimendan [26,27].

Intensive care physicians also use antibiotics, sedatives, inotropes, and blood products extensively. While necessary in many cases, there is an increasingly strong feeling that these agents are being overused, as many problems and complications are directly attributable to them. For example, excessive antibiotic use is related to the development of bacterial resistance and fungal overgrowth [28], while overuse of sedatives delays weaning from mechanical ventilation [29]. However, less consideration is paid to other effects of these drugs that could arguably be just as injurious, if not more so.

## Underlying Mechanisms for Why Our Treatments May Cause Harm

How can we explain, at the molecular level, the covert harm to the patient from standard drugs such as antibiotics, sedatives, and inotropes? The answer may lie in understanding the pathophysiological mechanisms underlying multiple organ failure, such as changes in immune and hormonal status and the role of the mitochondrion.

Over a billion years ago, a bacterium containing the oxygen-consuming respiratory chain is likely to have invaded the early eukaryotic cell. Most of the bacterial genetic information was subsequently transferred to the nucleus, transforming these bacterial symbionts into “slave” mitochondrial organelles. This provided a far more efficient system for using available energy sources and also protected the cell against the potentially toxic effects of oxygen. More than 90% of total body oxygen consumption is used to generate adenosine triphosphate (ATP) by the mitochondrial electron transport chain, and this, in turn, provides more than 90% of the body's power, the remainder coming from glycolysis.

**Figure 1:**
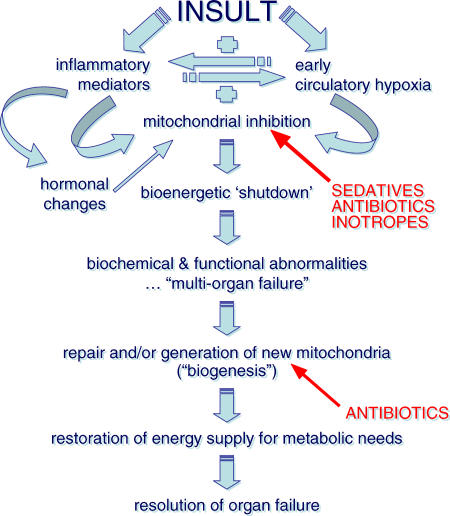
Hypothesis Explaining the Pathophysiology of Multiple Organ Failure Following Infection and Other Inflammatory Insults Antibiotics, sedatives, and inotropes may cause harm through inhibition of mitochondrial function. Antibiotics may also delay recovery by impeding mitochondrial regeneration.

An attractive hypothesis to explain the pathophysiology of multiple organ failure following infection and other inflammatory insults is a mitochondrial shutdown leading to “energy failure” and a consequent inability to drive the various metabolic processes that maintain normal cellular functioning (Figure 1). Inflammatory mediators released in considerable excess in sepsis, such as tumor necrosis factor and nitric oxide, are known to directly inhibit mitochondrial respiration. We and others have demonstrated this mechanism in septic patients and laboratory models [30–32]. The down-regulation of endocrine function seen in established sepsis, for example, the sick euthyroid syndrome, insulin resistance, and hypoleptinaemia, will also impinge on mitochondrial activity [33]. If the cell attempts to continue to function normally despite inadequate energy production, the resulting fall in adenosine triphosphate will trigger necrotic and apoptotic death pathways.

However, as this process is not immediate (it takes hours to days to develop fully) the cell has time to potentially adapt to this prolonged, life-threatening insult. It is likely to do so by entering a hibernation-like state. The impressive and almost total absence of cell death seen in organs that have “failed” biochemically and/or physiologically [34] lends credence to this hypothesis. Restoration of cellular function, and thus recovery from organ failure, must therefore depend upon repair of damaged mitochondria and/or production of new organelles, a process known as mitochondrial biogenesis.

## Harm from Antibiotics

The reason for this preamble is to emphasise the role of the systemic inflammatory response and the likely fundamental importance of the mitochondrion in the development of multiple organ failure, and also the mitochondrion's distant lineage but existing genetic linkage to bacteria. We use antibiotics to fight bacteria, and they are undoubtedly successful in many instances. Many of the antibiotic classes, such as the penicillins and cephalosporins, are bactericidal through cell-wall disruption, whereas other classes, such as chloramphenicol and aminoglycosides, act in a bacteriostatic manner by inhibiting protein synthesis. However, by virtue of their action, the cell-wall disrupters—in particular the cephalosporins—cause increased levels of endotoxin release from Gram-negative bacteria [35,36] and lipoteichoic acid and peptidoglycan release from Gram-positive bacteria [37,38]. This enhanced toxin release leads to significantly higher inflammatory mediator production. This may well explain the rapid clinical deterioration often seen in patients with sepsis after the first dose of cidal antibiotics, though long-term consequences remain unknown.

A delayed and potentially significant effect of antibiotics may be seen through their inhibition of mitochondrial activity and biogenesis. This inhibition has been shown in numerous in vitro studies, in which cell lines or isolated mitochondria have been incubated with antibiotics at concentrations equivalent to therapeutic blood levels. Significant decreases in respiratory enzyme activity and protein turnover have been found across a wide range of antibiotic classes [39–42]. Could our antibiotic therapy be thus accentuating the degree of sepsis and multiple organ dysfunction through increased inflammatory mediator release and mitochondrial depression? Could such therapy also be delaying recovery by impeding mitochondrial regeneration?

## Harm from Sedatives

Continuing on this theme, the major classes of sedative/anesthetic agents (opiates, benzodiazepines, propofol, barbiturates, and volatile anaesthetic agents) routinely used to enable mechanical ventilation in the operating theatre or intensive care unit all have effects on mitochondrial function in vitro [43–48]. In the case of propofol, these effects appear to be significantly enhanced in the presence of nitric oxide through formation of nitrosopropofol [45]. Thus, sepsis may potentially amplify the effects of this sedative agent on mitochondrial inhibition. This mechanism may explain the severe metabolic and physiological deterioration reported in children and adults receiving propofol [48].

All classes of sedative agents have also been shown to alter immune function in neutrophils, monocytes, and lymphocytes in vitro and to affect rates of apoptosis [49–54]. Immunosuppression (for example, assessed in monocytes by HLA-DR status) is well recognised in established sepsis and is related to worse outcomes [55,56]. The clinical significance of sedative drug actions on the immune response to critical illness remains unknown.

Certain sedatives are also known to affect hormonal status. The most striking example is the classic study by Watt and Ledingham, who sought an explanation for the sudden jump in mortality rates in their critically ill trauma patients: from 28% in those receiving opiates and benzodiazepines to 77% of those sedated with etomidate [57]. They showed a significant etomidate-induced depression of adrenal function that led to withdrawal of its use for medium- to long-term sedation in intensive care. Etomidate is, however, still frequently used as an induction agent for anaesthesia because of its cardiovascular stability. Unfortunately, this practice continues despite the fact that Absalom et al. have shown that a single dose of etomidate given before surgery in critically ill patients was sufficient to compromise adrenal function 24 hours later [58].

## Harm from Other Drugs and Interventions

Other drugs are known to affect hormone levels. Low-dose dopamine, which was a popular and subsequently disproved therapy for maintaining renal function, rapidly reduces serum prolactin levels [59]. Prolactin has immunostimulatory effects, and a low prolactin level has been associated with a worse outcome in septic mice [60]. The recognition that impaired adrenal function, as assessed by a subnormal rise in plasma cortisol to synthetic adrenocorticotropic hormone, was related to poor outcomes in septic shock [61] led to a multicentre trial that revealed survival benefit from early administration of hydrocortisone 50 mg four times daily [62]. However, the debate continues surrounding its contribution to the development of critical illness neuromyopathy and delayed weaning [63]. The sick euthyroid syndrome is likewise associated with worse outcomes [64] yet several drugs that affect thyroid function, such as amiodarone, are frequently used in critically ill patients.

These concerns can be replicated across virtually every therapy area in the critically ill.

### Mechanical ventilation.

A strategy of delivering low tidal volumes rather than the previously fashionable high tidal volumes during mechanical ventilation reduced mortality from 39% to 31% [13]; this change in strategy has been separately shown to also reduce both the local and systemic inflammatory response, presumably from lowering shear stresses within the lung [65].

### Immunonutrition.

A trial of immunonutrition had to be prematurely terminated after an interim analysis revealed a significant mortality increase in septic patients [66].

### Drotrecogin-alpha.

The Canadian Department of Health recently issued a safety alert on Drotrecogin-alpha (Xigris), the first licensed therapy for severe sepsis, after post-hoc analyses of trial data revealed an excess mortality in patients with single organ dysfunction who had received surgery within 30 days prior to study treatment [67].

### Blood transfusion.

Lowering the threshold for blood transfusion from 10 g/dl to 7 g/dl, and thus reducing transfusion requirements from an average of 5.6 ± 5.3 red-cell units per patient to 2.6 ± 4.1 units, reduced 30-day mortality rates from 23.3% to 18.7% [12]. In a recent retrospective analysis of three large trials of patients with acute coronary syndromes, Rao et al. reported a 3-fold increase in death and myocardial infarction rates in those who received a blood transfusion [68]. There may be an immunological reason underlying this apparent harm. Hebert et al. found significant reductions in mortality rates, post-transfusion fevers, and antibiotic use in patients who received leukoreduced blood transfusions, compared to historical controls who received “normal” blood [69]. It remains to be seen whether remaining blood constituents in leukocyte-depleted blood are able to also affect the immune response.

### Proton pump inhibitors.

A recent meta-analysis [70] comparing the use of proton pump inhibitors against either placebo or an H2-antagonist found a significant reduction in rebleeding and the need for surgical intervention. Yet despite this clear benefit, the trend in mortality was actually in the opposite direction. For studies of intravenous therapy, as is given to critically ill patients, the odds ratio for mortality was 1.22 (95% confidence interval 0.84–1.78).

## Conclusions

It should be immediately acknowledged that most of the above findings have been derived from relatively small patient studies or extrapolated from in vivo and in vitro laboratory studies. As with most aspects of medicine, there are contradictory results. Yet sufficient data exist to suggest that the possibility of insidious harm should not be lightly dismissed. The above litany of problems should also not be used as a reason to abandon current practices, but instead to stimulate discussion, refine their use, and to encourage trials designed to confirm or refute detriment.

Our concern is that neither the inclination nor the funding will be generally available to revisit accepted dogma. We will thus have to rely on a slowly evolving approach, where new therapies are compared with conventional treatments, or where a media-highlighted concern propels a certain strategy into the spotlight. This was the case, for example, with the use of albumin for fluid administration. A Cochrane meta-analysis suggested a 4% increase in mortality with albumin over crystalloid solutions [70]. The subsequent prospective randomised trial of 6,997 patients revealed no overall difference in mortality; intriguingly, subset analysis suggested benefit when used in sepsis but harm in head-injured patients [71].

## References

[pmed-0020167-b1] Lewis M (2004). The social history of the navy 1793–1815.

[pmed-0020167-b2] Beatty W (1805). List of wounded at Battle of Trafalgar. http://www.nationalarchives.gov.uk/pathways/battles/trafalgar/ts13a.htm.

[pmed-0020167-b3] Barrett CRB (1911). The 13th at Waterloo. http://www.pinetreeweb.com/waterloo.htm.

[pmed-0020167-b4] United States Civil War Center (2001). Statistical summary of America's major wars. http://www.cwc.lsu.edu/cwc/other/stats/warcost.htm.

[pmed-0020167-b5] Adams GW (1996). Doctors in blue: The medical history of the Union Army in the Civil War.

[pmed-0020167-b6] Armstrong GL, Conn LA, Pinner RW (1999). Trends in infectious disease mortality in the United States during the 20th century. JAMA.

[pmed-0020167-b7] Arias E (2004). United States life tables, 2002. Natl Vital Stat Rep.

[pmed-0020167-b8] Juni P, Nartey L, Reichenbach S, Sterchi R, Dieppe PA (2004). Risk of cardiovascular events and rofecoxib: Cumulative meta-analysis. Lancet.

[pmed-0020167-b9] Dellinger RP, Carlet JM, Masur H, Gerlach H, Calandra T (2004). Surviving Sepsis Campaign guidelines for management of severe sepsis and septic shock. Crit Care Med.

[pmed-0020167-b10] Gattinoni L, Brazzi L, Pelosi P, Latini R, Tognoni G (1995). A trial of goal-oriented hemodynamic therapy in critically ill patients. SvO_2_ Collaborative Group. N Engl J Med.

[pmed-0020167-b11] Hayes MA, Timmins AC, Yau EH, Palazzo M, Hinds CJ (1994). Elevation of systemic oxygen delivery in the treatment of critically ill patients. N Engl J Med.

[pmed-0020167-b12] Hebert PC, Wells G, Blajchman MA (1999). A multicenter, randomized, controlled clinical trial of transfusion requirements in critical care. Canadian Critical Care Trials Group. N Engl J Med.

[pmed-0020167-b13] The Acute Respiratory Distress Syndrome Network (2000). Ventilation with lower tidal volumes as compared with traditional tidal volumes for acute lung injury and the acute respiratory distress syndrome. N Engl J Med.

[pmed-0020167-b14] Northridge D (1996). Frusemide or nitrates for acute heart failure?. Lancet.

[pmed-0020167-b15] Nieminen MS, Bohm M, Cowie MR, Drexler H, Filippatos GS (2005). Executive summary of the guidelines on the diagnosis and treatment of acute heart failure: The Task Force on Acute Heart Failure of the European Society of Cardiology. Eur Heart J.

[pmed-0020167-b16] Cotter G, Metzkor E, Kaluski E (1998). Randomised trial of high-dose isosorbide dinitrate plus low-dose furosemide versus high-dose furosemide plus low-dose isosorbide dinitrate in severe pulmonary oedema. Lancet.

[pmed-0020167-b17] Clutter WE, Bier DM, Shah SD, Cryer PE (1980). Epinephrine plasma metabolic clearance rates and physiologic thresholds for metabolic and hemodynamic action in man. J Clin Invest.

[pmed-0020167-b18] Nikolaidis LA, Hentosz T, Doverspike A, Huerbin R, Stolarski C (2002). Catecholamine stimulation is associated with impaired myocardial O2 utilization in heart failure. Cardiovasc Res.

[pmed-0020167-b19] Bergmann M, Sautner T (2002). Immunomodulatory effects of vasoactive catecholamines. Wien Klin Wochenschr.

[pmed-0020167-b20] Arcaroli J, Yang KY, Yum HK, Kupfner J, Pitts TM (2002). Effects of catecholamines on kinase activation in lung neutrophils after hemorrhage or endotoxemia. J Leukoc Biol.

[pmed-0020167-b21] Levy B, Mansart A, Bollaert PE, Franck P, Mallie JP (2003). Effects of epinephrine and norepinephrine on hemodynamics, oxidative metabolism, and organ energetics in endotoxemic rats. Intensive Care Med.

[pmed-0020167-b22] Lyte M, Freestone PP, Neal CP, Olson BA, Haigh RD (2003). Stimulation of Staphylococcus epidermidis growth and biofilm formation by catecholamine inotropes. Lancet.

[pmed-0020167-b23] Freestone PP, Williams PH, Haigh RD, Maggs AF, Neal CP (2002). Growth stimulation of intestinal commensal Escherichia coli by catecholamines: A possible contributory factor in trauma-induced sepsis. Shock.

[pmed-0020167-b24] Cuffe MS, Califf RM, Adams KF (2002). Short-term intravenous milrinone for acute exacerbation of chronic heart failure: A randomized controlled trial. JAMA.

[pmed-0020167-b25] Cohn JN, Goldstein SO, Greenberg BH (1998). A dose-dependent increase in mortality with vesnarinone among patients with severe heart failure. N Engl J Med.

[pmed-0020167-b26] Follath F, Cleland JG, Just H, Papp JG, Scholz H (2002). Efficacy and safety of intravenous levosimendan compared with dobutamine in severe low-output heart failure (the LIDO study): A randomised double-blind trial. Lancet.

[pmed-0020167-b27] Coletta AP, Cleland JG, Freemantle N, Clark AL (2004). Clinical trials update from the European Society of Cardiology Heart Failure meeting: SHAPE, BRING-UP 2 VAS, COLA II, FOSIDIAL, BE
TACAR, CASINO and meta-analysis of cardiac resynchronisation therapy. Eur J Heart Fail.

[pmed-0020167-b28] Rybak MJ (2004). Resistance to antimicrobial agents: An update. Pharmacotherapy.

[pmed-0020167-b29] Kress JP, Pohlman AS, O'Connor MF, Hall JB (2000). Daily interruption of sedative infusions in critically ill patients undergoing mechanical ventilation. N Engl J Med.

[pmed-0020167-b30] Brealey D, Brand M, Hargreaves I, Heales S, Land J (2002). Association between mitochondrial dysfunction and severity and outcome of septic shock. Lancet.

[pmed-0020167-b31] Brealey D, Karyampudi S, Jacques TS, Novelli M, Stidwill R (2004). Mitochondrial dysfunction in a long-term rodent model of sepsis and organ failure. Am J Physiol Regul Integr Comp Physiol.

[pmed-0020167-b32] Boulos M, Astiz ME, Barua RS, Osman M (2003). Impaired mitochondrial function induced by serum from septic shock patients is attenuated by inhibition of nitric oxide synthase and poly(ADP-ribose) synthase. Crit Care Med.

[pmed-0020167-b33] Singer M, De Santis V, Vitale D, Jeffcoate W (2004). Multiorgan failure is an adaptive, endocrine-mediated, metabolic response to overwhelming systemic inflammation. Lancet.

[pmed-0020167-b34] Hotchkiss RS, Swanson PE, Freeman BD, Tinsley KW, Cobb JP (1999). Apoptotic cell death in patients with sepsis, shock, and multiple organ dysfunction. Crit Care Med.

[pmed-0020167-b35] Prins JM, van Deventer SJ, Kuijper EJ, Speelman P (1994). Clinical relevance of antibiotic-induced endotoxin release. Antimicrob Agents Chemother.

[pmed-0020167-b36] Lepper TK, Held EM, Schneider E, Bolke H, Gerlach M (2002). Clinical implications of antibiotic-induced endotoxin release in septic shock. Intensive Care Med.

[pmed-0020167-b37] Heer C, Stuertz K, Reinert RR, Mader M, Nau R (2000). Release of teichoic and lipoteichoic acids from 30 different strains of Streptococcus pneumoniae during exposure to ceftriaxone, meropenem, quinupristin/dalfopristin, rifampicin and trovafloxacin. Infection.

[pmed-0020167-b38] van Langevelde P, van Dissel JT, Ravensbergen E, Appelmelk BJ, Schrijver IA (1998). Antibiotic-induced release of lipoteichoic acid and peptidoglycan from Staphylococcus aureus: quantitative measurements and biological reactivities. Antimicrob Agents Chemother.

[pmed-0020167-b39] Riesbeck K, Bredberg A, Forsgren A (1990). Ciprofloxacin does not inhibit mitochondrial functions but other antibiotics do. Antimicrob Agents Chemother.

[pmed-0020167-b40] Wilkie D (1977). Mitochondrial biogenesis: Inhibitors of mitochondrial protein synthesis. Mol Cell Biochem.

[pmed-0020167-b41] Mela-Riker LM, Widener LL, Houghton DC, Bennett WM (1986). Renal mitochondrial integrity during continuous gentamicin treatment. Biochem Pharmacol.

[pmed-0020167-b42] Tune BM, Hsu CY (1990). The renal mitochondrial toxicity of beta-lactam antibiotics: In vitro effects of cephaloglycin and imipenem. J Am Soc Nephrol.

[pmed-0020167-b43] Hosein EA, Lapalme M, Sacks B, Wiseman-Distler M (1979). Biphasic changes in rat brain mitochondrial membrane structure and enzyme activity after acute opiate administration to rats. Biochem Pharmacol.

[pmed-0020167-b44] Colleoni M, Costa B, Gori E, Santagostino A (1996). Biochemical characterization of the effects of the benzodiazepine, midazolam, on mitochondrial electron transfer. Pharmacol Toxicol.

[pmed-0020167-b45] Stevanato R, Momo F, Marian M, Rigobello MP, Bindoli A (2002). Effects of nitrosopropofol on mitochondrial energy-converting system. Biochem Pharmacol.

[pmed-0020167-b46] Kupriyanov VV, Lakomkin VL, Korchazhkina OV, Stepanov VA, Steinschneider AY (1991). Cardiac contractile function, oxygen consumption rate and cytosolic phosphates during inhibition of electron flux by amytal—a 31P-NMR study. Biochim Biophys Acta.

[pmed-0020167-b47] Miro O, Barrientos A, Alonso JR, Casademont J, Jarreta D (1999). Effects of general anaesthetic procedures on mitochondrial function of human skeletal muscle. Eur J Clin Pharmacol.

[pmed-0020167-b48] Vasile B, Rasulo F, Candiani A, Latronico N (2003). The pathophysiology of propofol infusion syndrome: A simple name for a complex syndrome. Intensive Care Med.

[pmed-0020167-b49] Larsen B, Hoff G, Wilhelm W, Buchinger H, Wanner GA (1998). Effect of intravenous anesthetics on spontaneous and endotoxin-stimulated cytokine response in cultured human whole blood. Anesthesiology.

[pmed-0020167-b50] Song HK, Jeong DC (2004). The effect of propofol on cytotoxicity and apoptosis of LPS-treated mononuclear cells and lymphocytes. Anesth Analg.

[pmed-0020167-b51] Brand JM, Frohn C, Luhm J, Kirchner H, Schmucker P (2003). Early alterations in the number of circulating lymphocyte subpopulations and enhanced proinflammatory immune response during opioid-based general anesthesia. Shock.

[pmed-0020167-b52] Helmy SA, Al-Attiyah RJ (2001). The immunomodulatory effects of prolonged iv infusion of propofol versus midazolam in critically ill surgical patients. Anaesthesia.

[pmed-0020167-b53] Kelbel I, Koch T, Weber A, Schiefer HG, van Ackern K (1999). Alterations of bacterial clearance induced by propofol. Acta Anaesthesiol Scand.

[pmed-0020167-b54] Delogu G, Antonucci A, Moretti S, Marandola M, Tellan G (2004). Oxidative stress and mitochondrial glutathione in human lymphocytes exposed to clinically relevant anesthetic drug concentrations. J Clin Anesth.

[pmed-0020167-b55] Docke WD, Randow F, Syrbe U, Krausch D, Asadullah K (1997). Monocyte deactivation in septic patients: Restoration by IFN-gamma treatment. Nat Med.

[pmed-0020167-b56] Lekkou A, Karakantza M, Mouzaki A, Kalfarentzos F, Gogos CA (2004). Cytokine production and monocyte HLA-DR expression as predictors of outcome for patients with community-acquired severe infections. Clin Diagn Lab Immunol.

[pmed-0020167-b57] Watt I, Ledingham IM (1984). Mortality amongst multiple trauma patients admitted to an intensive therapy unit. Anaesthesia.

[pmed-0020167-b58] Absalom A, Pledger D, Kong A (1999). Adrenocortical function in critically ill patients 24 h after a single dose of etomidate. Anaesthesia.

[pmed-0020167-b59] Bailey AR, Burchett KR (1997). Effect of low-dose dopamine on serum concentrations of prolactin in critically ill patients. Br J Anaesth.

[pmed-0020167-b60] Zellweger R, Zhu XH, Wichmann MW, Ayala A, DeMaso CM (1996). Prolactin administration following hemorrhagic shock improves macrophage cytokine release capacity and decreases mortality from subsequent sepsis. J Immunol.

[pmed-0020167-b61] Annane D, Sebille V, Troche G, Raphael JC, Gajdos P (2000). A 3-level prognostic classification in septic shock based on cortisol levels and cortisol response to corticotropin. JAMA.

[pmed-0020167-b62] Annane D, Sebille V, Charpentier C, Bollaert PE, Francois B Effect of treatment with low doses of hydrocortisone and fludrocortisone on mortality in patients with septic shock. JAMA.

[pmed-0020167-b63] De Jonghe B, Sharshar T, Lefaucheur JP (2002). Paresis acquired in the intensive care unit: A prospective multicenter study. JAMA.

[pmed-0020167-b64] Leon-Sanz M, Lorente JA, Larrodera L, Ros P, Alvarez J (1997). Pituitary-thyroid function in patients with septic shock and its relation with outcome. Eur J Med Res.

[pmed-0020167-b65] Ranieri VM, Suter PM, Tortorella C, De Tullio R, Dayer JM (1999). Effect of mechanical ventilation on inflammatory mediators in patients with acute respiratory distress syndrome: A randomized controlled trial. JAMA.

[pmed-0020167-b66] Bertolini G, Iapichino G, Radrizzani D, Facchini R, Simini B (2003). Early enteral immunonutrition in patients with severe sepsis: Results of an interim analysis of a randomised multicentre clinical trial. Intensive Care Med.

[pmed-0020167-b67] Health Canada (2005). Notice to hospitals: Health Canada endorsed important safety information on Xigris. http://www.hc-sc.gc.ca/hpfb-dgpsa/tpd-dpt/xigris_nth_e.pdf.

[pmed-0020167-b68] Rao SV, Jollis JG, Harrington RA, Granger CB, Newby LK (2004). Relationship of blood transfusion and clinical outcomes in patients with acute coronary syndromes. JAMA.

[pmed-0020167-b69] Hebert PC, Fergusson D, Blajchman MA (2003). Clinical outcomes following institution of the Canadian universal leukoreduction program for red blood cell transfusions. JAMA.

[pmed-0020167-b70] Leontiadis GI, Sharma VK, Howden CW (2005). Systematic review and meta-analysis of proton pump inhibitor therapy in peptic ulcer bleeding. BMJ.

[pmed-0020167-b71] Human albumin administration in critically ill patients: Systematic review of randomised controlled trials. Cochrane Injuries Group Albumin Reviewers. BMJ.

[pmed-0020167-b72] Finfer S, Bellomo R, Boyce N, French J, Myburgh J (2004). A comparison of albumin and saline for fluid resuscitation in the intensive care unit. N Engl J Med.

